# On Common Neuroses

**Published:** 1893-03

**Authors:** 


*On Common Neuroses.* By JAMES FREDERIC GOODHART, M.D., F.R.C.P. Pp. 128. London: H. K. LEWIS. 1892.

Dr. Goodhart has here reprinted in book-form the three lectures which he delivered before the Harveian Society in 1891. These lectures are characterised by sound judgment and extensive clinical experience, and are further rendered attractive reading by the author's wit and power of terse writing. They are, moreover, thought-compelling, and abound in useful advice as to the treatment of a class of cases which often tax the physician's resources to the uttermost, and in which common-sense and thoughtfulness are indispensable factors to success in treatment. Anything that Dr. Goodhart has to say on the subject is deserving of careful study, and we are sure that those who have read these lectures in the *Lancet* will be glad of the opportunity that is afforded by this neat little volume to possess them in a more convenient form.

